# Polysaccharides L900/2 and L900/3 isolated from *Lactobacillus rhamnosus* LOCK 0900 modulate allergic sensitization to ovalbumin in a mouse model

**DOI:** 10.1111/1751-7915.12606

**Published:** 2017-02-06

**Authors:** Sabina Górska, Martin Schwarzer, Dagmar Srutkova, Petra Hermanova, Ewa Brzozowska, Hana Kozakova, Andrzej Gamian

**Affiliations:** ^1^ L. Hirszfeld Institute of Immunology and Experimental TherapyPolish Academy of SciencesWeigla 1253‐114WrocławPoland; ^2^ Institute of MicrobiologyLaboratory of GnotobiologyAcademy of Sciences of the Czech Republic v. v. i.549 22Novy HradekCzech Republic

## Abstract

Here, we compared the abilities of polysaccharides L900/2 and L900/3, which were previously isolated from *Lactobacillus rhamnosus* LOCK 0900, to modulate the immune response to bystander antigens in a mouse model of ovalbumin (OVA) sensitization. *In vivo*, both polysaccharides reduced the levels of OVA‐specific IgE, IgE‐dependent basophil degranulation and IgG2a antibodies, but had no effect on the levels of OVA‐specific IgA or IgG1. Interestingly, both polysaccharides triggered recall cellular responses with distinct properties. L900/3 significantly suppressed the OVA‐induced upregulations of IL‐4, IL‐5, IL‐10 and IL‐13 in re‐stimulated spleen cells and mesenteric lymph nodes. Our findings support and expand on our previous *in vitro* studies by demonstrating that polymer L900/3 might modulate the Th1/Th2 balance and could be a promising candidate molecule for preventing allergic sensitization.

## Introduction

The intestinal epithelium is constantly exposed to a multitude of foreign materials (e.g. food antigens, pathogens, toxins and commensal bacteria) that can harm or benefit the organism. The intestinal immune system needs to find a balance between protective immune responses and antigen tolerance. Deregulation of these crucial processes can lead to an inappropriate immune response to food, triggering a food allergy characterized by excessive activations of mast cells, basophils and T helper type 2 (Th2) cells (Vighi *et al*., 2008). In recent decades, the prevalence of allergic diseases has consistently increased in industrialized countries. The exact causes of this enhancement are not known. Several hypotheses have been proposed, including theories based on epidemiological evidence of a reduced microbial burden in early childhood, and those related to the exaggerated hygiene of the typical Western lifestyle (known as the ‘hygiene hypothesis’).

It is well known that the gut microbiota plays a crucial role in establishing tolerance to food antigens. This supports the increasing interest in the use of probiotic bacteria to prevent allergy development. Accumulating laboratory and clinical evidence support the notion that oral probiotics may prevent the development of allergic sensitization and/or disease (Toh *et al*., 2012). However, there have been some contradictory results, and the beneficial effects of probiotics appear to depend on the use of specific strains and the timing of treatment. The action mechanisms of probiotic bacteria are multifaceted and not yet fully understood. It appears that each probiotic bacterium may have specific effects on the host, involving a variety of effector signals, cell types and receptors (Kim *et al*., 2008; Rupa *et al*., 2011; Schabussova *et al*., 2011; Zuercher *et al*., 2012). Although probiotics have been shown to confer some benefit in treating allergies, the risks may outweigh the benefits in people predisposed to adverse events, such as immunosuppressed individuals. Clinical reports have shown that these otherwise beneficial bacteria can spread to the blood in at‐risk individuals, causing a life‐threatening infection known as sepsis (Liong, 2008; Whelan and Myers, 2010). Clearly, additional research is needed to fully explore the role of probiotics in immune system development and their use in preventing or treating allergic diseases. Moreover, we need to clarify the biological role of different bacterial components, including the surface antigens, and whether such components could be exploited in place of whole bacteria. The relevant surface antigens include the polysaccharides (PS), which are biopolymers that may be released into the environment or remain attached to the bacterial cell wall. PS are extremely diverse in their compositions and have been widely applied in various food and pharmaceutical industries. Some evidence suggests that they have health‐promoting properties (Ciszek‐Lenda *et al*., 2011; Fanning *et al*., 2012; Górska *et al*., 2014, 2016).

The aim of this study was to determine the ability of polysaccharides L900/2 and L900/3 isolated from *Lactobacillus rhamnosus* LOCK 0900 to modulate the immune response to bystander antigens in a mouse model of ovalbumin (OVA) sensitization. We previously reported our comprehensive analysis of the chemical structure and molecular mass of polysaccharides L900/2 and L900/3 and showed that they could actively change the immune response to a bacterium other than their parent strain. We hypothesized that the high‐molecular‐mass PS L900/2 may act as a regulatory molecule, whereas the small polymer, L900/3, may enhance the proinflammatory response (Górska *et al*., 2014).

## Results

**Figure 1:**
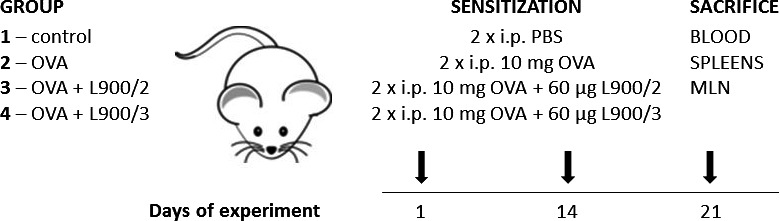
Systemic sensitization of BALB/c mice to ovalbumin (OVA). Experimental design: eight‐week‐old female BALB/c mice were divided into four groups (4–6 mice per group) and intraperitoneally sensitized with PBS, 10 mg of OVA, 10 mg of OVA + 60 μg of L900/2 or 10 mg of OVA + 60 μg of L900/3, with alum (Al(OH)_3_), on days 1 and 14. Experiment was repeat experiments, each with four to six mice per group.

We previously reported a comprehensive chemical analysis of polysaccharides L900/2 or L900/3 (Górska *et al*., 2014). To evaluate whether these polysaccharides could influence the systemic cellular and humoral responses to OVA sensitization, we treated mice with OVA with or without polysaccharides, using two administrations with a 14 day interval between them (Fig. 1).

### Effect of L900/2 and L900/3 on total IgE, IgA and OVA‐specific antibody levels in serum

**Figure 2:**
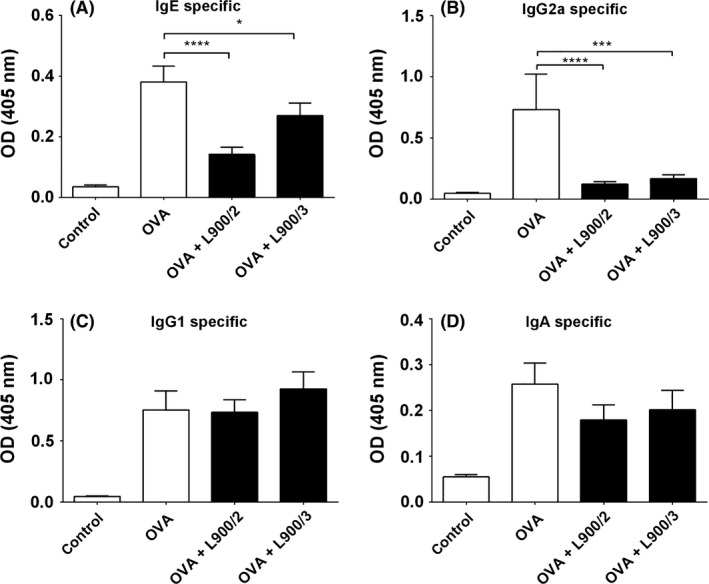
Levels of OVA‐specific IgE (A), IgG2a (B), IgG1 (C) and IgA (D) antibodies in serum, as measured by ELISA. Pooled values of two repeat experiments, each with four to six mice per group, are expressed as means ± SEM; *P <* *0.05, ***P <* *0.001, ****P < 0.0001.

**Figure 3:**
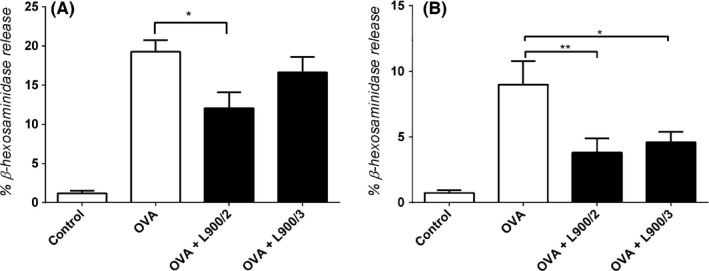
Functional IgE in serum diluted at 1:270 (A) or 1:810 (B) was measured based on the OVA‐mediated release of β‐hexosaminidase from rat basophil leukaemia cells. Pooled values of two repeat experiments, each with four to six mice per group, are expressed as means ± SEM; *P <* *0.05, **P <* *0.01.

**Figure 4:**
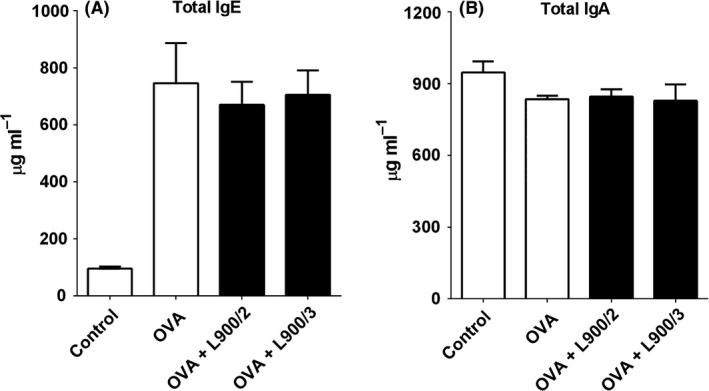
Levels of total IgE (A) and IgA (B) in serum, as measured by ELISA. Pooled values of two repeat experiments, each with four to six mice per group.

We compared the abilities of L900/2 and L900/3 to modulate allergic reactions in the well‐established mouse model of allergic OVA sensitization. The application of polysaccharides reduced the serum levels of OVA‐specific IgE and Th1‐related IgG2a compared to those in mice treated with OVA alone (Fig. 2A and B), suggesting that the PS prevented allergic sensitization. Neither PS was found to influence the OVA‐specific IgG1 or IgA antibody response in serum (Fig. 2C and D). Both polysaccharides reduced the IgE‐dependent basophil degranulation observed in response to OVA (Fig. 3), further confirming that these treatments decreased OVA‐specific IgE. However, the productions of total IgA and IgE, as the serum levels of these antibodies, were comparable across all three groups (Fig. 4).

### Effect of L900/2 and L900/3 on cellular responses

**Figure 5:**
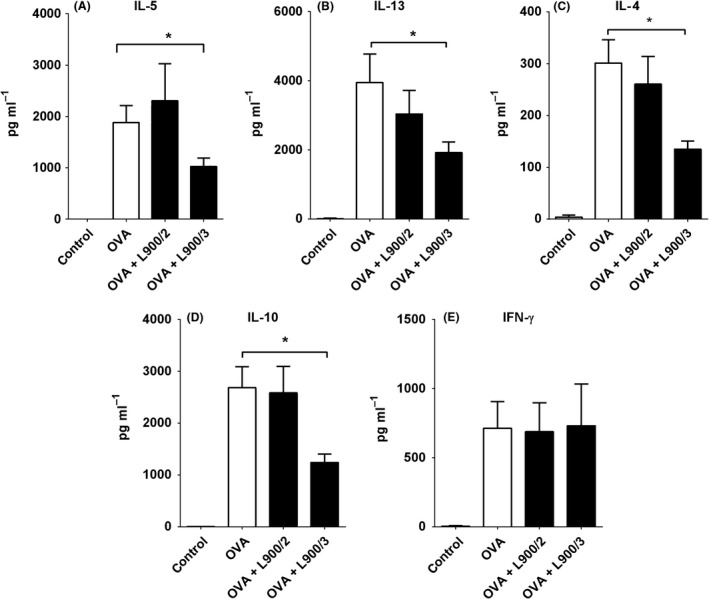
Influence of polysaccharides L900/2 and L900/3 on OVA‐induced cytokine production in splenocytes, as measured using a MILLIPLEX cytokine panel. Pooled values of two repeat experiments, each with four to six mice per group, are expressed as means ± SEM; *P < 0.05.

**Figure 6:**
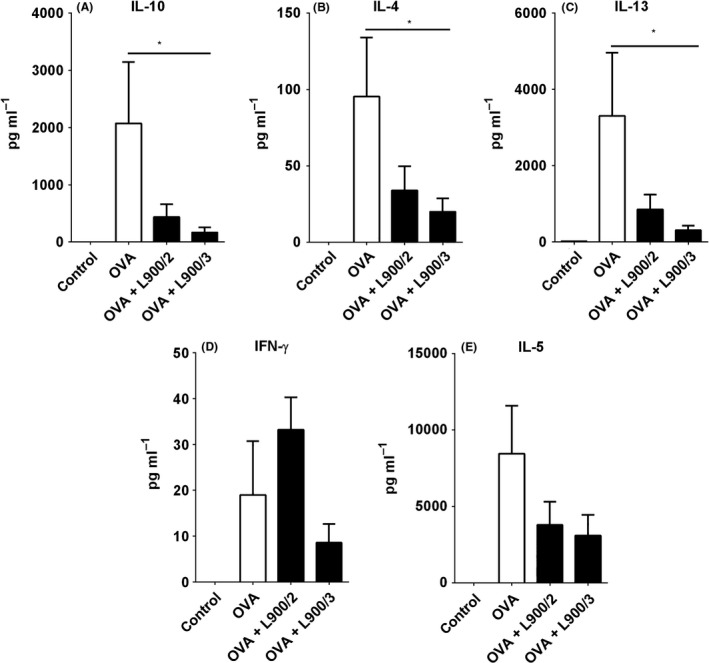
Influence of polysaccharides L900/2 and L900/3 on OVA‐induced cytokine production in MLN, as measured using a MILLIPLEX cytokine panel. Pooled values of two repeat experiments, each with four to six mice per group, are expressed as means ± SEM; *P < 0.05.

We examined OVA‐specific recall responses in spleen and MLN cells of sensitized mice. In spleen cell cultures, polysaccharide L900/3 suppressed the productions of the Th2‐related cytokines, IL‐5, IL‐13 and IL‐4, whereas L900/2 did not significantly change the cytokine levels compared to those observed in OVA‐treated mice (Fig. 5A–C). Polymer L900/3 also reduced the level of the regulatory molecule, IL‐10, whereas L900/2 had no effect on this factor (Fig. 5D). The level of the pro‐inflammatory molecule, IFN‐ɣ, was not affected by either treatment (Fig. 5E). Treatment with polysaccharide L900/2 was associated with downregulation of IL‐17, whereas L900/3 treatment tended to increase this interleukin, although not to a significant degree (*P *>* *0.05, data not shown). In MLN cell cultures, L900/3 significantly suppressed the productions of IL‐10, IL‐4 and IL‐13 (Fig. 6A–C) and tended to non‐significantly decrease the levels of IFN‐ɣ and IL‐5 to non‐significant degrees (Fig. 6D and E). In the same cells, L900/2 tended to decrease the levels of IL‐10, IL‐4, IL‐5 and IL‐13 to non‐significant degrees (*P *>* *0.05), but significantly increased the level of IFN‐ɣ (Fig. 6D).

### Differentiation of CD4^+^CD25^+^Foxp3^+^ T cells

Finally, we isolated CD4^+^ T cells from mouse MLN and spleens and used fluorescence‐activated cell sorting to quantify the number of such cells that expressed intracellular Foxp3 in mice of each group. However, we found that treatment with polysaccharide L900/2 or L900/3 did not significantly affect the levels of Foxp3^+^ CD4^+^ T cells in MLNs or spleens compared to those in control samples (Fig. S1).

## Discussion

This study explored for the first time the potential ability of polysaccharides L900/2 and L900/3 isolated from *L*. *rhamnosus* LOCK 0900 to prevent allergic sensitization *in vivo*. Our previous structural studies of these PS, including classical chemical analysis, nuclear magnetic resonance spectroscopy, mass spectrometry and *in vitro* immunomodulatory studies (e.g. TLR recognition, NOD2 receptor recognition and dendritic cell stimulation), showed appreciable differences between the two (Górska *et al*., 2014). We previously proposed that the longer, high‐molecular‐weight polymer, L900/2, may act as a regulatory molecule, whereas the smaller L900/3 might function as a stimulatory molecule. To evaluate whether the differences identified *in vitro* are reflected *in vivo*, we tested the effects of the two polymers during OVA sensitization of mice.

Both polymers significantly reduced the levels of OVA‐specific IgE (associated with a Th2 phenotype) and IgG2a (required for the participation of Th1 cells), but had no effect on OVA‐specific IgA or IgG1 (required for the participation of Th2 cells). These results are not completely surprising, given that IgG1 antibodies have a much longer half‐life than IgG2a antibodies, and the levels of antigen‐specific immunoglobulins build up over time upon repeated sensitizations, leading to the long‐term persistence of memory B cells (Israel *et al*., 1996). Previous studies using whole bacteria obtained similar results following the administration of probiotics in different allergy models. In mice with OVA‐induced food allergy, for example, *Bifidobacterium lactis/bifidum* and *Lactobacillus acidophilus* suppressed the productions of OVA‐specific IgE, IgG_1_ and IgA (Kim *et al*., 2008). Ohno *et al*. (2005) demonstrated that *Bifidobacterium bifidum* G9‐1 significantly and powerfully reduced the serum levels of total and antigen‐specific IgE without decreasing antigen‐specific IgG1 and increasing of the specific IgG2a. Likewise, Schabussova *et al*. (2012) showed that the levels of allergen‐specific IgE and IgG were unaltered by *Lactobacillus paracasei* NCC 2461 in a mouse model of birch pollen allergy. However, other reports have suggested that some probiotic strains believed to promote Th1‐type immunoresponses can suppress the productions of both IgE and IgG1 (Ishida *et al*., 2003; Schwarzer *et al*., 2013).

Interestingly, we found that the two tested polysaccharides exhibited differences in their systematic cellular responses. For example, polymer L900/3 significantly suppressed the productions of IL‐4, IL‐5, IL‐10 and IL‐13, but increased the level of IL‐17, whereas polysaccharide L900/2 failed to alter IL‐4, IL‐5, IL‐10 or IL‐13, but decreased the level of IL‐17. The prevention of allergy is known to be associated with the downregulations of IL‐4, IL‐5, IL‐13 and IL‐10 (Schabussova and Wiedermann, 2008). Under our experimental conditions, the level of IFN‐ɣ was not altered in mice treated with L900/2 or L900/3. These results emphasize that different polysaccharides may have different effects on the anti‐OVA response and that the efficacy of a polysaccharide‐based intervention is strongly dependent on the chemical features of the polymer.

Based on our findings that L900/3 significantly decreased Th2‐related cytokine secretion and IL‐10 production, we speculate that L900/3 suppresses OVA sensitization *via* a mechanism that does not involve the suppression of Th1‐related cytokines or a shift of the Th1/Th2 balance towards Th1‐dominant immunity. Our data are consistent with those from whole‐bacterium studies showing that allergy prevention is associated with suppression of both Th1 and Th2 systemic responses. For example, *Lactobacillus paracasei* NCC 2461 and *Bifidobacterium longum* NCC 3001 suppressed local Th1 and Th2 responses and the productions of IL‐5, IFN‐ɣ and IL‐10 in re‐stimulated spleen cells (Schabussova *et al*., 2011). Neonatal monocolonization of germ‐free mice with *Bifidobacterium longum* subspecies *longum* CCM 7952 suppressed the production of both Th1‐ and Th2‐associated cytokines in spleen cell cultures induced with the allergen, Bet v 1 (Schwarzer *et al*., 2013). In another study, eight common *Lactobacillus* strains were studied with respect to the ability of the murine gut mucosa to induce cytokine production in response to a parenterally administered antigen; the results indicated that *L. reuteri* induced proinflammatory and Th1 cytokines, whereas *L. casei* tended to induce IL‐10 and IL‐4 (Maassen *et al*., 2000). *B*. *bifidum* G9‐1 was found to induce IFN‐ɣ and IL‐12 from splenocytes and to reduce the OVA‐stimulated productions of IL‐4 and IL‐5 *in vitro* (Ohno *et al*., 2005). Likewise, *L*. *rhamnosus* GG was associated with reductions in antigen‐specific Th2 cytokines, IFN‐ɣ and IL‐10 in allergen re‐stimulated spleen cells (Blumer *et al*., 2007). Moreover, the oral administration of an immunostimulatory DNA sequence from *B. longum* was found to suppress Th2 immune responses in mice and inhibit IgE production *in vitro* (Takahashi *et al*., 2006). Despite the above findings, however, other studies have shown that certain probiotics had no significant effect on the Th1 or Th2 cell responses to allergens (Christensen *et al*., 2002). This indicates that multiple biological pathways can contribute to regulating the aberrant immune responses of an allergic response.

To the best of our knowledge, this is the first study to show that a component isolated from a probiotic bacterium, namely polysaccharide L900/3, could prevent OVA sensitization. This suggests that L900/3 could be a promising candidate for the management of allergic symptoms. Further studies are needed to identify the mechanisms through which this polymer acts to control and/or improve allergic hypersensitivity.

## Experimental procedures

### Animals

Female BALB/c mice (8 weeks of age) were maintained in IVC cages (Tecniplast, Buguggiate, Italy) under conventional specific pathogen‐free conditions with a 12:12‐h light–dark cycle and a sterile diet (Altromin Spezialfutter GmbH & Co. KG, Lage, Germany). The animal experiments were approved by the committee for the protection and use of experimental animals of the Institute of Microbiology, The Czech Academy of Sciences, v. v. i., and by the 1st Local Committee for Experiments with the Use of Laboratory Animals, Wroclaw, Poland (no. 38/2012).

### Bacterial strains

*Lactobacillus rhamnosus* LOCK 0900 was obtained from the Pure Culture Collection of the Technical University of Lodz, Poland (LOCK). Strains were stored at −75°C in MRS broth (Biocorp, Warsaw, Poland) supplemented with 20% glycerol. For use, cells were cultured for 48 h in MRS broth (Biocorp), centrifuged and washed with sterile phosphate‐buffered saline (PBS).

### Isolation and purification of polysaccharides L900/2 and L900/3

The isolation and purification of polysaccharides L900/2 and L900/3 were performed as previously described (Górska *et al*., 2014). In brief, lyophilized bacteria were extracted with trichloroacetic acid and the polysaccharides were ethanol precipitated from the supernatant, collected by centrifugation (pellet fraction) and suspended in water. The dialysed and lyophilized material was exhaustively digested with DNase (Sigma, Saint Louis, Missouri, USA), RNase (Sigma, Saint Louis, Missouri, USA) and protease (Sigma, Saint Louis, Missouri, USA). The digest was dialysed against water, lyophilized and purified by ion‐exchange chromatography follow by gel filtration using an FPLC system (Amersham Pharmacia Biotech, Uppsala, Sweden). The obtained material was checked by NMR spectroscopy Bruker 600 MHz Avance III spectrometer ((Bruker, BioSpin, Fällanden, Switzerland), and the level of LPS contamination was detected with a Limulus amoebocyte lysate assay (PyroGene recombinant factor C endotoxin detection assay; Lonza, Basel, Switzerland). Finally, the average molecular mass was determined by gel permeation chromatography (GPC).

### Experimental design

Twenty BALB/c mice were divided into four groups and treated with PBS, OVA, OVA + L900/2 or OVA + L900/3 (4–6 mice per group). Mice were sensitized by intraperitoneal (i.p.) injections of 10 mg of OVA (Worthington, Lakewood, NJ, USA) or 10 mg of OVA + 60 μg of each polysaccharide adsorbed to 100 μl of aluminium hydroxide (Alum, Serva, Germany) in a final volume of 200 μl, on days 1 and 14. Control mice received 100 μl PBS and 100 μl aluminium hydroxide. On day 7 after the last immunization, the mice were euthanized by cervical dislocation. Blood was collected, and serum samples were stored at −40°C until analysis. Mesenteric lymph nodes (MLN, pooled per group) and spleens were aseptically removed and prepared for cytokine determination. All experiments were repeated twice using four to six mice per group each time.

### Serum levels of OVA‐specific IgE, IgA, IgG1 and IgG2a, and basophil release assay

OVA‐specific serum IgG1, IgG2a, IgE and IgA levels were determined by ELISA. The 96‐well microtitre plates were coated with OVA (5 μg ml^−1^). Serum samples were diluted 1:10,000 for IgG1, 1:100 for IgG2a, 1:10 for IgE and 1:10 for IgA. Rat anti‐mouse IgG1, IgG2a, IgE and IgA antibodies (2 μl ml^−1^; Pharmingen, San Diego, CA USA) were applied, followed by peroxidase‐conjugated mouse anti‐rat IgG antibodies (1:2000; Jackson Immuno Labs, West Grove, PA, USA). Antibody levels were quantified based on optical densities.

The allergen‐specific IgE levels in sera were quantified by analysing the degranulation of rat basophil leukaemia (RBL‐2H3) cells (Wiedermann *et al*., 2001). RBL‐2H3 cells were plated in 96‐well tissue culture plates (4 × 10^4^ cells per well) and sensitized by incubation with mouse serum (diluted 1:270 or 1:810) for 2 h. The cells were washed, OVA (0.6 mg ml^−1^) was added, and the cells were incubated for 30 min at 37°C for degranulation. Supernatants were recovered and incubated with 4‐methylumbelliferyl‐N‐acetyl‐β‐D‐glucosaminide (Sigma‐Aldrich, St Louis, MO, USA), and β‐hexosaminidase was analysed using an Infinite M200 fluorescence microplate reader (λex:360 nm/λem:465 nm; Tecan Group, Grodig, Austria). The results are reported as the percentage of total β‐hexosaminidase released from cells after disruption with 1% Triton X‐100.

### Total IgA and IgE responses

The serum levels of total IgA and IgE were measured using a mouse IgA and IgE ELISA quantification kit (Bethyl, Montgomery, TX, USA) according to the manufacturer's instructions. The serum samples were diluted 1:800 for measurement of IgA and 1:10 for measurement of IgE.

### Quantification of cytokine production

Spleen cells and pooled MLN cell suspensions were cultured in 48‐well flat‐bottom plates at a concentration of 5 × 10^6^ cells in 500 ml of complete RPMI 1640. Cells were cultivated with/without OVA (10 mg well^−1^) re‐stimulation at 37°C under 5% CO_2_ for 48 h. Supernatants were collected and stored at −20°C until analysis. The levels of IL‐4, IL‐5, IL‐10, IL‐13, IL‐17 and IFN‐ɣ were determined using a Mouse Cytokine/Chemokine Multiplex Immunoassay (Millipore, Milford, MA, USA) according to the manufacturer's instructions. The results were analysed with a Luminex 200 System (Bio‐Rad Laboratories, Irvine, CA, USA). Values are expressed as pg ml^−1^.

### Flow cytometry

Single‐cell suspensions of spleen or MLN were stained for regulatory T cells using the Foxp3 Staining Buffer Set (eBioscience, San Diego, CA, USA) as previously described (Golias *et al*., 2012). Cells were analysed using a FACSCalibur flow cytometer (Becton‐Dickinson,Wilson, NC, USA), and the obtained data were analysed with the flowjo 7.6.2 software (TreeStar, Ashland, OR, USA).

### Statistics

Between‐group comparisons were analysed using one‐way analysis of variance (ANOVA) followed by the Student's *t*‐test, using the graphpad prism 5.04 (GraphPad, San Diego, CA, USA). Data are expressed as the mean ± SEM. In all tests, *P* values of < 0.05 were taken as reflecting a statistically significant difference.

## Conflict of interest

The authors declare that no conflict of interest exists.

## Supporting information


**Fig. S1.** Foxp3 expression in the mesenteric lymph nodes and spleens of OVA‐treated mice.
