# Supplementary material for: Polysaccharides L900/2 and L900/3 isolated from Lactobacillus rhamnosus LOCK 0900 modulate allergic sensitization to ovalbumin in a mouse model
Source: Microb Biotechnol. 2017 Feb 6;10(3):586–93. doi: 10.1111/1751-7915.12606 (PMC5404188; doi:10.1111/1751-7915.12606)
Supplement: Supplementary file 1 — Fig. S1. Foxp3 expression in the mesenteric lymph nodes and spleens of OVA‐treated mice. [file MBT2-10-586-s001.docx]

**Fig. S1**. **Foxp3 expression in mesenteric lymph nodes and spleen of OVA-treated mice.**
